# In vitro identification and in vivo metabolic profiling of chemical constituents in *Moringa oleifera* seeds

**DOI:** 10.1016/j.fochx.2025.102899

**Published:** 2025-08-08

**Authors:** Jiahong Wang, Juan Cao, Hao Wang, Yudie Zhang, Li Jiang, Jiaohan Zhan, Yanxiu Sun, Yiyang Du, Tingxu Yan, Ying Jia, Bosai He

**Affiliations:** a School of Functional Food and Wine, Shenyang Pharmaceutical University, Wenhua Road 103, Shenyang 110016, China; b School of Clinical Pharmacy, Shenyang Pharmaceutical University, Shenyang 110016, China; c School of Pharmacy, Shenyang Pharmaceutical University, Wenhua Road 103, Shenyang 110016, China

**Keywords:** *Moringa oleifera* seeds, Chemical composition analysis, Metabolism in vivo, Pharmacokinetics

## Abstract

*Moringa oleifera* seeds (MOS), recognized as a nutritionally valuable food, are rich in diverse bioactive compounds and widely utilized for disease prevention and adjunctive therapy. However, their in vitro chemical constituents and in vivo blood-absorbed/metabolized components remain underexplored. This study pioneered the application of UPLC-Q-Exactive Orbitrap-MS to characterize MOS-derived compounds in vitro and in vivo, concurrently elucidating major constituents' fragmentation pathways via mass spectrometry. In vitro, 81 chemical components of MOS were preliminarily characterized. 38 blood-borne components, comprising 11 prototypes and 27 metabolites, were identified in serum samples from MOS-treated rats. A validated LC-MS/MS method was developed to quantify the pharmacokinetics of key bioactive compounds (6-gingerol, vanillin, and eugenol) in rat plasma. These findings establish a theoretical foundation for clarifying MOS composition, evaluating its dietary applications, and guiding further exploration of its pharmacological potential.

## Highlights

• First UPLC-Q-Exactive MS analysis of MOS chemical constituents in vitro and in vivo.
• 81 MOS compounds identified, including glucosinolates, flavonoids, and phenolic acids.
• 38 serum components (11 prototypes, 27 metabolites) characterized in MOS-treated rats.
• Elucidated mass fragmentation patterns of key MOS components for metabolic research.

## 1 Introduction

*Moringa oleifera* Lam. (Moringa), a perennial tropical deciduous tree of the Moringaceae family, is indigenous to India and Africa. It is colloquially termed the “drumstick tree” due to its elongated, triangular-shaped pods. (Jaglan et al., 2025). The seeds of this species possess exceptional nutritional value. In vitro analyses confirm MOS as a rich source of proteins (36 %), lipids (38.7 %), vitamins (notably vitamin E: 100 mg/752 g dried seeds), minerals (magnesium: 100 mg/635 g; copper: 2.100 mg/5 g), and glucosinolates. (Oyeyinka & Oyeyinka, 2018). MOS additionally exhibits significant pharmacological properties, including antioxidant and hypoglycemic activities (Oboh, Oyeleye, Akintemi, & Olasehinde, 2018; Sharma, Wichaphon, & Klangpetch, 2020). These bioactive attributes have facilitated the global cultivation of MOS as a valuable cash crop.

MOS contains diverse bioactive constituents: all essential and semi-essential amino acids, alongside numerous non-essential amino acids (Aderinola, Fagbemi, Enujiugha, Alashi, & Aluko, 2018). Phytochemical investigations haincludingfied multiple polyphenolic compounds in MOS, including flavonoids, phenolic acids, tannins, etc., exhibiting a total phenolic content (TPC) ranging from 4581 to 4953 mg/100 g (Govardhan Singh, Negi, & Radha, 2013). Experimental studies mechanistically link these phenolic constituents to MOS's anti-inflammatory, antioxidant, and hypoglycemic effects (Oboh et al., 2018; Sharma et al., 2020). Glucosinolates emerge as a predominant phytochemical class in MOS, constituting 2.5–20 % of its chemical composition (Chen et al., 2019), characterized by structural homogeneity with acetylated isomers (Sayed, Omar, Emam, & Farag, 2022). In vivo studies validate their anti-inflammatory, anticancer, and gut microbiota-modulating properties (L. Huang, Yuan, & Wang, 2020; Jaja-Chimedza et al., 2018), positioning MOS glucosinolates as promising candidates for natural therapeutics and functional foods.

Over 100 chemical constituents have been documented in MOS. However, characterization predominantly employs Gas Chromatography-Tandem Mass Spectrometry (GC–MS/MS) or conventional phytochemical isolation. Given the prevalence of non-volatile and thermally labile constituents in MOS, High-Performance Liquid Chromatography-Mass Spectrometry (HPLC-MS) offers advantages by enabling room-temperature separation while preserving structural integrity (X.-F. Huang et al., 2024), coupled with high resolution for macromolecules (Sayed et al., 2022; Xu, Chen, & Guo, 2019).

Quadrupole-Orbitrap high-resolution mass spectrometry (Q-Exactive Orbitrap-MS) has emerged as a premier analytical platform in phytochemical research, distinguished by superior mass resolution, exceptional mass accuracy, extended mass range, and broad dynamic range (Wołoszyn-Durkiewicz & Myśliwiec, 2019). This technology enables simultaneous detection of ionic species via full-scan acquisition with auto-triggered MS/MS fragmentation in dual ionization modes, effectively mitigating matrix interference inherent in traditional herbal analyses (F. Jiang et al., 2024; Liu, Li, Fu, Zhang, & Tang, 2024). When integrated with Ultra-Performance Liquid Chromatography (UPLC), the system enhances chromatographic resolution and accelerates compound identification, establishing a robust framework for systematic phytochemical profiling(Ogurtsova et al., 2022). This investigation employed an optimized pharmacognostic workflow to conduct dual-phase characterization of MOS aqueous extracts—encompassing comprehensive in vitro chemical mapping and preliminary in vivo metabolite tracking. This integrated strategy facilitates holistic constituent identification while providing foundational data for elucidating pharmacodynamic mechanisms and metabolic pathways.

## 2 Materials and methods

### 2.1 Materials and reagents

MOS were procured from commercial suppliers in Fujian and Guangdong provinces between June 2021 and September 2022. HPLC-grade methanol and acetonitrile were obtained from Thermo Fisher Scientific (Waltham, MA, USA). Formic acid was acquired from Chengdu Kolon Chemical Co. (Chengdu, China). Ultra-pure water was generated using a Millipore Milli-Q® water purification system (Shanghai, China). The following reference standards were purchased from Chengdu Krom Biotechnology Co., Ltd. (Chengdu, China) with certified purities:Moringin (benzyl 4-((2*S*,3*R*,4*S*,5*S*,6*R*)-3,4,5-trihydroxy-6-(hydroxymethyl)tetrahydro-2H- pyran-2-yloxy)isothiocyanate; ≥98 %); 6-Gingerol ((5*S*)-5-hydroxy-1-(4-hydroxy-3- methoxyphenyl)decan-3-one; ≥98 %); Syringaldehyde (4-hydroxy-3,5- dimethoxybenzaldehyde; ≥98 %); Vanillin (4-hydroxy-3-methoxybenzaldehyde; ≥98 %); p-Coumaric acid ((E)-3-(4-hydroxyphenyl)prop-2-enoic acid; ≥98 %); 7-Hydroxycoumarin (7-hydroxy-2H-chromen-2-one; ≥98 %); Vitexin (apigenin-8-C-β -D-glucopyranoside; ≥98 %); Glucomoringin (benzyl 4-((2*S*,3*R*,4*S*,5*S*,6*R*)-3,4,5- trihydroxy-6-(hydroxymethyl) tetrahydro-2H-pyran-2-yloxy)-1-thioglucopyranosate; ≥97 %).

### 2.2 Animals

Male adult Sprague-Dawley (SD) rats (220–250 g) were procured from Liaoning Longevity Company, qualified number: SCXK (liao) 2020–0001, No. 210726221101071034/1137. They were housed in SPF Animal Laboratory Center, Shenyang Pharmaceutical University (License No. SCXK (Liao) 2020), where the temperature was maintained between 22 and 25 °C, the humidity was kept constant at 40–65 %, and a 12-h light-dark cycle was maintained, and were given free diet and water. Cages and daily drinking water were sterilized using high pressure. All animal experiments were operated strictly according to Laboratory Animal Management in China, conducted under the guidance of Laboratory Animal Care and Use, and approved by the Laboratory Animal Welfare and Ethics Committee of Shenyang Pharmaceutical University (approval no. SYPU-IACUC-S2022–08.04-201).

### 2.3 Sample preparation

#### 2.3.1 Test solution preparation

MOS kernels were homogenized and sieved (80 mesh). The powder (accurately weighed) underwent aqueous extraction (1:10, *w*/*v*) using deionized water. Cell disruption was performed for 2 min followed by ultrasonication (45 min). This extraction procedure was repeated twice. Combined filtrates were concentrated under reduced pressure using a rotary evaporator. The concentrate was diluted appropriately, filtered through a 0.22-μm organic microporous membrane (discarding initial 1 mL filtrate), and the subsequent filtrate served as the test solution.

#### 2.3.2 Reference solution preparation

Individual stock solutions of moringin, 6-gingerol, syringaldehyde, vanillin, p-coumaric acid, 7-hydroxycoumarin, vitexin, and glucomoringin were prepared by dissolving accurately weighed reference standards in methanol within separate 5-mL volumetric flasks. Aliquots of each stock solution were quantitatively transferred to a 10-mL volumetric flask and diluted to volume with methanol, yielding the mixed reference solution.

#### 2.3.3 MOS intragastric solution preparation

Sieved MOS powder (80 mesh) was accurately weighed and extracted with deionized water (1:10, *w*/*v*) using identical disruption and ultrasonication parameters (2 min disruption, 45 min ultrasonication × 2). Combined filtrates were concentrated under reduced pressure to obtain a standardized solution (2.12 g/mL equivalent raw material). The solution was stored at −20 °C until administration.

### 2.4 Preparation of serum samples

#### 2.4.1 Serum collection

Blood samples (1 mL) were collected from the retro-orbital plexus of six male SD rats into 1.5 mL centrifuge tubes at predefined intervals (0 [pre-dose], 0.5, 1, 2, 4, and 6 h) following intragastric administration of MOS concentrate (3 mL; 25.44 g raw material equivalent/kg body weight). Rats were fasted for 12 h with ad libitum water access prior to dosing. Collected blood samples were clotted for 45 min at ambient temperature, then centrifuged at 4000 rpm (10 min, 4 °C). Serum supernatants were aliquoted and stored at −80 °C pending analysis. Post-experiment, rats were euthanized via cervical dislocation under deep anesthesia induced by intraperitoneal sodium pentobarbital injection.

#### 2.4.2 Serum pretreatment

Aliquots (200 μL) of thawed serum underwent protein precipitation with three volumes of methanol. After vortex-mixing (30 s) and vigorous agitation (3 min), samples were centrifuged (10,000 rpm, 5 min, 4 °C). Supernatants were transferred and evaporated to dryness under nitrogen stream (35 °C). Residues were reconstituted in 100 μL methanol, vortex-mixed (3 min), sonicated to complete dissolution, and centrifuged (10,000 rpm, 5 min, 4 °C). Resulting supernatants (2 μL injection volume) were subjected to LC-MS/MS analysis.

### 2.5 UPLC-Q-Exactive Orbitrap-MS analysis

Chromatographic separation was performed on a Thermo Vanquish UPLC system (Thermo Fisher Scientific, USA) equipped with an ACQUITY UPLC® HSS T3 column (100 mm × 2.1 mm, 1.7 μm; Waters, Milford, MA, USA). The mobile phase consisted of (A) acetonitrile and (B) 0.1 % aqueous formic acid. Separation was achieved under the following gradient conditions at 0.3 mL/min flow rate and 35 °C column temperature: 0–0.8 min, 2 % A; 0.8–2.8 min, 2 % → 70 % A; 2.8–5.6 min, 70 % → 90 % A; 5.6–6.4 min, 90 % → 100 % A; 6.4–8.0 min, 100 % A; 8.0–8.1 min, 100 % → 2 % A; 8.1–10.0 min, 2 % A. Injection volume was 5 μL.

Mass spectrometric detection employed a Q-Exactive Orbitrap mass spectrometer (Thermo Fisher Scientific, USA) with electrospray ionization (ESI) source. Operational parameters were as follows: Nebulization gas: High-purity nitrogen. Collision gas: High-purity helium. Scan range: *m*/*z* 100–1000. Acquisition mode: Full MS/dd-MS^2^. Spray voltage: ±3.0 kV (positive/negative modes). Stepped normalized collision energies: 20 %, 40 %, 60 %. Desolvation temperatures: 310 °C (positive mode), 350 °C (negative mode). Capillary temperature: 320 °C.

### 2.6 Pharmacokinetic study of MOS

#### 2.6.1 Analytical conditions

Chromatographic separation employed an ACQUITY UPLC® HSS C18 column (2.1 mm × 50 mm, 1.8 μm; Waters, Milford, MA, USA) at 0.4 mL/min flow rate and 30 °C. The mobile phase consisted of (C) 0.1 % aqueous formic acid and (D) methanol. Gradient elution: 0–2 min, 90 % → 45 % C (10 % → 55 % D); 2–5 min, 45 % → 10 % C (55 % → 90 % D); 5–6 min, 10 % C (90 % D); 6–6.1 min, 10 % → 90 % C (90 % → 10 % D); 6.1–8 min, 90 % C (10 % D). Injection volume was 4 μL. Mass spectrometric conditions matched those described in Section 2.5.

#### 2.6.2 Standard solution preparation

Stock solutions of 6-gingerol, vanillin, and syringaldehyde were prepared in methanol. Serial dilutions yielded standard solutions at these concentrations: 6-Gingerol: 35.7, 178.5, 357, 892.5, 1785, 3570, 8930, 17850 ng/mL. Vanillin: 17.72, 88.6, 177.2, 443, 886, 4430, 8860 ng/mL. Syringaldehyde: 40.56, 202.8, 405.6, 1014, 2028, 4056, 10140, 20280 ng/mL. Quality control (QC) samples were similarly prepared at low, medium, and high concentrations: 6-Gingerol: 89.2, 1389, 13390 ng/mL. Vanillin: 44.3, 664.5, 6645 ng/mL. Syringaldehyde: 101.4, 1521, 15210 ng/mL.

#### 2.6.3 Internal standard solution

Sulfamethoxazole (IS; 1.008 mg/mL stock in methanol) was diluted to 10 ng/mL working solution. Aliquots were stored at 4 °C.

#### 2.6.4 Calibration standards

Pooled blank plasma (180 μL) from six rats was spiked with 20 μL serial standard solutions. Calibration standards were prepared as per Section 2.6.5 with these concentrations: 6-Gingerol: 3.57, 17.85, 35.7, 89.3, 178.5, 357, 893, 1785 ng/mL. Vanillin: 1.772, 8.86, 17.72, 44.3, 88.6, 177.2, 443, 886 ng/mL. Syringaldehyde: 4.056, 20.28, 40.56, 101.4, 202.8, 405.6, 1014, 2028 ng/mL.

#### 2.6.5 Plasma sample processing

Rats received MOS concentrate (2.5 mL/rat; 32.2 g crude equivalent/kg) via gavage. Blood (0.5 mL) was collected from the orbital plexus into heparinized tubes at: 0 (pre-dose), 0.083, 0.25, 0.5, 0.75, 1, 1.5, 2, 4, 6, 8, 12, and 24 h post-dosing. Plasma was separated by centrifugation (10,000 rpm, 5 min, 4 °C).

#### 2.6.6 LC-MS/MS method validation

##### 2.6.6.1 Selectivity

Pooled blank plasma from six rats was processed without internal standard (Section 2.6.5) and analyzed (4 μL injection). Fortified plasma samples (spiked with mixed standards and IS) and 0.5-h post-dose plasma samples were similarly processed (2 μL injection). Chromatograms were evaluated for interference at analyte retention times.

##### 2.6.6.2 Calibration curve and **t**he lower limit of quantification

Calibration standards (duplicates) were processed per Section 2.6.5. Linear regression (weighting factor: 1/x^2^) of peak area ratios (analyte/IS) versus nominal concentrations established calibration curves. The lower limit of quantification (LLOQ) was determined as the lowest concentration with ≤20 % relative error and ≤ 20 % relative standard deviation (RSD).

##### 2.6.6.3 Precision and accuracy

QC samples (low, medium, high; *n* = 6 per level) were analyzed across three validation days. Intra-day precision (RSD) and accuracy (% nominal) were determined from single-day replicates. Inter-day values were derived from pooled three-day data.

##### 2.6.6.4 Extraction recovery and matrix effect

Recovery: Fortified pre-extraction samples (n = 6 per QC level) were compared with post-extraction spiked samples. Matrix effect: Post-extraction fortified samples were compared with neat standards. Matrix factor (MF) was calculated as: MF = (peak area of post-extraction spike) / (peak area of neat standard).

##### 2.6.6.5 Stability

QC samples (low: ∼3 × LLOQ; high: ∼ULOQ; *n* = 3) were assessed for: Room temperature stability (24 h). Post-preparation stability (12 h, 4 °C). Freeze-thaw stability (3 cycles, −80 °C). Long-term stability (15 d, −80 °C).

##### 2.6.6.6 Sample analysis and pharmacokinetics

Plasma concentrations were quantified against daily calibration curves. QC samples (≥5 % of total injections) were interspersed throughout analytical batches. Pharmacokinetic parameters (Tₘₐₓ, Cₘₐₓ, AUC₀₋ₜ, AUC₀₋∞, t₁/₂) were derived via non-compartmental analysis (DAS 2.0). Data are expressed as mean ± SD (GraphPad Prism 8.0; SPSS 22.0 for statistical comparisons).

### 2.7 Data processing

By referring to HMDB, METLIN, LipidMaps, and other databases and related literature, the structural formulas of various chemical components and the cleavage rules of compounds in MOS were collected and sorted out. Xcalibur 4.1 software was used to process the mass spectral data, and the molecular formula was calculated by high-resolution mass spectrometry. The possible compound cleavage rules were speculated based on the existing literature information. The mass spectral deviation range ppm ≤ 5 × 10–6 was preliminary speculated based on the literature for other compounds. The first reported compounds were identified by mass spectrometry according to their cleavage regularity.

## 3 Results and discussion

### 3.1 Composition characteristics of MOS aqueous extract

**Fig. 1:**
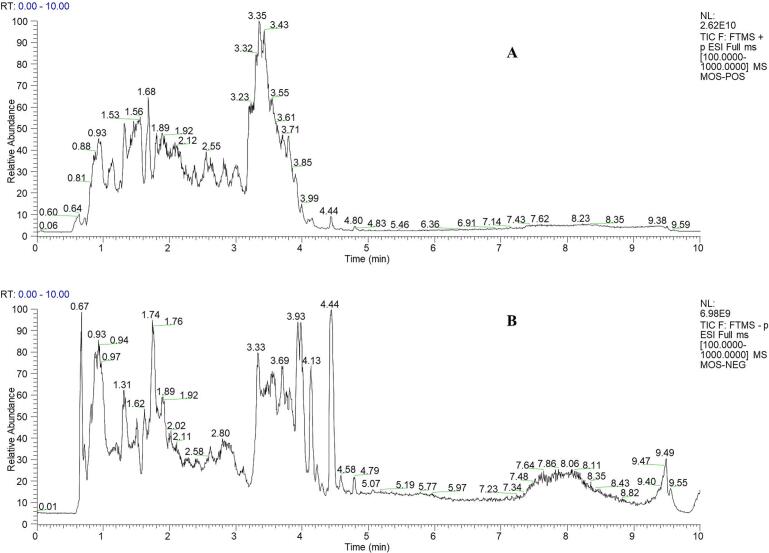
UPLC-Q-Extractive chromatograms. (A) TIC diagram of MOS in positive ion mode. (B) TIC diagram of MOS in negative ion mode.

Comprehensive phytochemical profiling of MOS aqueous extract was conducted via UPLC-Q-Exactive Orbitrap-MS. Total ion chromatograms (TIC) and MS/MS spectra acquired in dual ionization modes (Fig. 1, Table 1) facilitated compound characterization. Chromatographic peak processing through Xcalibur 4.1 software enabled annotation against established databases and literature references, utilizing both molecular formula matching and diagnostic fragment ion analysis. Eighty-one constituents were identified, categorized as: 5 glucosinolates, 7 alkaloids, 11 flavonoids (including glycosylated forms), 3 anthraquinones, 4 coumarins, 20 phenolic acids, 1 sesquiterpenoid, 1 polysaccharide, 15 organic acids, 8 amino acids, and 6 miscellaneous compounds (Table 1).

**Table 1:** List of compounds identified of MOS in positive and negative ion mode.

No.	TR	Theoretical *m/z* (Da)	Measured *m/z*(Da)	Error (ppm)	MS/MS	Predicted Formula	Compounds	Type
1	0.46	131.0462	131.0411	−3.89	87.0460，113.0255	C_4_H_8_N_2_O_3_	L-Asparagine	[M-H]^−^
2	0.57	131.0714	131.0720	4.57	85.0305	C_6_H_12_O_3_	2-Hydroxycaproic acid	[M-H]^−^
3	0.67	177.0546	177.0550	2.14	121.1016，135.0812	C_10_H_8_O_3_	7-Methoxycoumarin	[M + H]^+^
4	0.76	175.1190	175.1187	−1.71	175.1188，116.0705，70.0651，60.0556	C_6_H_14_N_4_O_2_	L-Arginine	[M + H]^+^
5	0.80	120.0655	120.0651	−3.33	74.0600，102.0217，56.0497	C_4_H_9_NO_3_	L-Threonine	[M + H]^+^
6	0.82	146.0459	146.0466	4.79	102.0570，128.0362	C_5_H_9_NO_4_	L-Glutamic acid	[M-H]^−^
7	0.92	195.0510	195.0514	2.05	75.0097，99.0097，129.0202	C_6_H_12_O_7_	Gluconic acid	[M-H]^−^
8	0.93	105.0193	105.0196	2.85	59.0151，72.9943，75.0099	C_3_H_6_O_4_	Glycolic acid	[M-H]^−^
9	0.96	191.0561	191.0567	3.14	111.0096，87.0088，85.0304，173.0096	C_7_H_12_O_6_	Quinic acid	[M-H]^−^
10	1.00	115.0037	115.0044	6.08	71.0139，115.0138	C_4_H_4_O_4_	Fumaric acid	[M-H]^−^
11	1.04	209.0303	209.0308	2.39	85.0306，129.0203	C_6_H_10_O_8_	Saccharic acid	[M-H]^−^
12	1.08	115.0037	115.0042	4.34	71.0149	C_4_H_4_O_4_	Fumaric acid	[M-H]^−^
13	1.09	341.1089	341.1088	−2.93	161.0463，101.0245，89.0244，71.0139，59.0139	C_12_H_22_O_11_	Trehalose	[M-H]^−^
14	1.10	133.0142	133.0150	5.66	115.0037，71.0139，72.9931，89.0244	C_4_H_6_O_5_	L-Malic acid	[M-H]^−^
15	1.14	287.0550	287.0544	−0.64	153.0179，258.0519	C_15_H_10_O_6_	Kaempferol	[M + H]^+^
16	1.19	191.0197	191.0202	2.62	85.0305，111.0097，129.0203，154.9993	C_6_H_8_O_7_	Isocitric acid	[M-H]^−^
17	1.21	173.0092	173.0096	2.31	111.0098	C_6_H_6_O_6_	Aconitic acid	[M-H]^−^
18	1.25	147.1128	147.1122	−4.08	84.0442，129.0180	C_6_H_14_N_2_O_2_	l-Lysine	[M + H]^+^
19	1.25	205.0972	205.0978	3.20	188.0702，146.0598	C_11_H_12_N_2_O_2_	L-tryptophan	[M + H]^+^
20	1.26	164.0473	163.0478	3.06	145.0413，119.0358	C_9_H_8_O_3_	p-Coumaric acid	[M-H]^−^
21	1.27	191.0197	191.0204	3.66	111.0097，87.0098，85.0305	C_6_H_8_O_7_	Citric acid	[M-H]^−^
22	1.38	106.0499	106.0495	−3.77	60.0334，87.0438	C_3_H_7_NO_3_	l-Serine	[M-H]^−^
23	1.52	182.0812	182.0805	−3.57	165.0544，147.0439，136.0755，123.0439，119.0490	C_9_H_11_NO_3_	L-Tyrosine	[M + H]^+^
24	1.54	117.0193	117.0198	4.27	116.0718，99.0087，73.0295，72.0173	C_4_H_6_O_4_	Succinic acid	[M-H]^−^
25	1.56	177.0546	177.0548	0.16	121.0650，105.0699	C_10_H_8_O_3_	4-Methylumbelliferone	[M + H]^+^
26	1.65	147.0299	147.0306	4.76	87.0097，85.0305，129.0202	C_5_H_8_O_5_	Citiamalate	[M-H]^−^
27	1.75	424.0377	424.0383	3.93	74.9919，96.9610，195.0341，259.0129，274.9907	C_14_H_19_NO_10_S_2_	Glucosinalbin	[M-H]^−^
28	1.88	219.1743	219.1747	0.35	175.1191，116.0707，70.0497，60.0557	C_15_H_22_O	Nootkatone	[M + H]^+^
29	1.93	199.0600	199.0608	−1.00	171.0804，153.0659	C_9_H_10_O_5_	Ethyl gallate	[M + H]^+^
30	2.05	243.0623	243.0635	2.29	110.0257，200.0569，216.0727	C_9_H_12_N_2_O_6_	Uridine	[M-H]^−^
31	2.42	492.1534	492.1556	4.58	107.0491，184.0425，185，0456	C_20_H_29_NO_11_S	N-[4-(β-L-rhamnopyranosyl)benzyl]-1-O-α-d-glucopyranosyl-thiocarboxamide	[M + H]^+^
32	2.48	586.0906	586.0921	2.59	96.9611，344.0807，259.0132，274.9905，290.9866，74.9585	C_20_H_29_NO_15_S_2_	3-hydroxyglucomoringin	[M-H]^−^
33	2.67	474.1428	474.1423	−1.79	107.0490，166.0313,328.0843,408.1104	C_20_H_27_NO_0_S	Moringin glucoside	[M + H]^+^
34	3.11	333.0671	331.0672	3.71	125.0253	C_13_H_16_O_10_	Galloyl-hexose	[M-H]^−^
35	3.15	299.0772	299.0751	−3.39	137.0523,93.0357	C_13_H_16_O_8_	p-hydroxybenzoic acid glucoside	[M-H]^−^
36	3.15	408.0428	408.0424	3.51	96.9610，80.9663，74.9920，328.0861	C_14_H_19_NO_9_S_2_	Glucotropaeolin	[M-H]^−^
37	3.15	328.1391	328.1372	−5.60	292.1173,264.1227,208.0968,166.0860,97.0283,69.0334	C_15_H_21_NO_7_	methyl N-[[4-(3,4,5-trihydroxy-6-methyloxan-2-yl)oxyphenyl]methyl]carbamate isomer 1	[M + H]^+^
38	3.19	359.0984	359.0975	−2.50	197.0459，179.0355	C_15_H_20_O_10_	Methoxypolygoacetophenoside	[M-H]^−^
39	3.25	315.0722	315.0721	3.15	153.0565，108.0227	C_13_H_16_O_9_	Protocatechuic acid hexose	[M-H]^−^
40	3.26	515.1195	515.1210	2.91	179.0355，135.0460	C_25_H_24_O_12_	Di-O-Caffeoylquinic acid isomer	[M-H]^−^
41	3.30	570.0957	570.0962	0.09	96.9611，74.9910，328.0859，259.0128，274.9902	C_20_H_29_NO_14_S_2_	Glucomoringin	[M-H]^−^
42	3.32	344.1162	344.1162	−4.82	107.0493，238.0712	C_15_H_21_NO_6_S	Niazinin A	[M + H]^+^
43	3.32	353.0878	353.0880	−0.05	191.0567，179.0356，135.0461	C_16_H_18_O_9_	Caffeoylquinic acid isomer 1	[M-H]^−^
44	3.32	328.1391	328.1387	−1.15	292.1172,264.1227,208.0966,166.0860,97.0283,69.0335	C_15_H_21_NO_8_	methyl N-[[4-(3,4,5-trihydroxy-6-methyloxan-2-yl)oxyphenyl]methyl]carbamate isomer 2	[M + H]^+^
45	3.36	153.0193	153.0200	4.57	109.0303，123.0460	C_7_H_6_O_4_	Protocatechuic acid	[M-H]^−^
46	3.40	339.0721	339.0722	3.37	177.0120，133.0303	C_15_H_16_O_9_	Esculin	[M-H]^−^
47	3.42	337.0929	337.0931	0.60	119.0511，163.0483，173.0460，191.0567	C_16_H_18_O_8_	p-Coumaroyl Qiiinic acid isomer 1	[M-H]^−^
48	3.43	353.0878	353.0878	0.02	191.0566，179.0355，135.0459	C_16_H_18_O_9_	Caffeoylquinic acid isomer 2	[M-H]^−^
49	3.45	179.0350	179.0356	3.91	135.0461	C_9_H_8_O_4_	Caffeic acid	[M-H]^−^
50	3.47	367.1035	367.1038	3.90	193.0511，134.0383，173.0461	C_17_H_20_O_9_	Feruloylquinic acid isomer 1	[M-H]^−^
51	3.51	447.0946	447.0940	1.34	269.1034，327.0512	C_22_H_16_N_4_O_7_	Carminic acid	[M-H]^−^
52	3.54	337.0929	337.0933	4.71	119.0511，163.0483，173.0460，191.0567	C_16_H_18_O_8_	p-Coumaroyl Qiiinic acid isomer 2	[M-H]^−^
53	3.54	353.0878	353.0882	1.13	191.0567，179.0355，135.0460	C_16_H_18_O_9_	Caffeoylquinic acid isomer 3	[M-H]^−^
54	3.55	137.0244	137.0251	1.81	109.0301，93.0355	C_7_H_6_O_3_	3,4-Dihydroxybenzaldehyde	[M-H]^−^
55	3.58	612.1062	612.1069	2.93	74.9919，96.9610，195.0336，259.0131，274.9898	C_22_H_31_NO_15_S_2_	4-(2′-acetyl-α-L-rhamnosyloxy)-benzyl isothiocyanate isomer	[M-H]^−^
56	3.59	367.1035	367.1028	1.31	193.0511，134.0383，173.0461	C_17_H_20_O_9_	Feruloylquinic acid isomer 2	[M-H]^−^
57	3.60	298.1285	298.1298	1.25	107.0490，136.0758，152.0704	C_14_H_19_NO_6_	Marumoside A	[M + H]^+^
58	3.61	431.0984	431.0990	3.70	311.0564，283.0615，269.0457	C_21_H_20_O_10_	Apigenin glucoside isomer 1	[M-H]^−^
59	3.62	463.0882	463.0889	1.77	300.0277，301.0344，271.0251，255.0303	C_21_H_20_O_12_	Isoquercitrin	[M-H]^−^
60	3.62	593.1512	593.1517	1.57	285.0403，284.0328，255.0302，227.0353	C_27_H_30_O_15_	Kaempferol-3-O-rutinoside/Kaempferol-O-hexose-rhamnose	[M-H]^−^
61	3.65	549.0886	549.0890	2.72	300.0276，301.0344，271.0250，255.0301	C_24_H_22_O_15_	Quercetin malonylglucoside isomer1	[M-H]^−^
62	3.69	447.0933	447.0938	1.65	255.0302，284.0328，285.0394，227.0354	C_21_H_20_O_11_	Astragalin	[M-H]^−^
63	3.70	367.1035	367.1032	2.40	193.0511，134.0383，173.0461	C_17_H_20_O_9_	Feruloylquinic acid isomer 3	[M-H]^−^
64	3.71	477.1038	477.1043	3.14	314.0340，271.0249，243.0300	C_22_H_22_O_12_	Isorhamnetin-3-O-Glucoside	[M-H]^−^
65	3.74	353.0878	353.0877	−0.28	191.0566，179.0356，135.0460	C_16_H_18_O_9_	Caffeoylquinic acid isomer 4	[M-H]^−^
66	3.76	337.0929	337.0926	2.36	119.0511，163.0483，173.0460，191.0567	C_16_H_18_O_8_	p-Coumaroyl Qiiinic acid isomer 3	[M-H]^−^
67	3.76	609.1461	609.1469	3.18	300.0277，301.0344，271.0251，255.0303	C_27_H_30_O_16_	Rutin	[M-H]^−^
68	3.81	431.0984	431.0986	3.22	311.0563，283.0614，269.0454	C_21_H_20_O_10_	Apigenin glucoside isomer 2	[M-H]^−^
69	3.82	431.0984	431.0987	3.27	269.0454，311.0563，413.0881	C_21_H_20_O_10_	Emodin-8-O-glucoside	[M-H]^−^
70	3.83	463.0882	463.0888	3.69	300.0276，301.0344，271.0250，255.0301	C_21_H_20_O_12_	Quercetin glycoside isomer 1/Isoquercitrin	[M-H]^−^
71	3.88	187.0976	187.0981	2.67	97.0669，125.0981，123.0825，126.1014	C_9_H_16_O_4_	Azelaic acid	[M-H]^−^
72	3.88	433.1124	433.1129	0.26	283.0594，313.0698	C_21_H_20_O_10_	Vitexin	[M + H]^+^
73	3.90	151.0401	151.0408	4.63	92.0267，121.0303，136.0174	C_8_H_8_O_3_	Vanillin	[M-H]^−^
74	4.10	201.1132	201.1138	2.98	139.1137，183.1032	C_10_H_18_O_4_	Sebacic acid	[M-H]^−^
75	4.12	137.0244	137.0250	4.37	93.0355	C_7_H_6_O_3_	4-Hydroxybenzoic acid	[M-H]^−^
76	4.26	179.0703	179.0704	0.18	161.0958，133.1010，105.0697	C_10_H_10_O_3_	3-Methoxycinnamic acid	[M + H]^+^
77	4.42	312.0900	312.0888	−1.21	107.0490，206.0477	C_14_H_17_NO_5_S	Moringin	[M + H]^+^
78	4.57	183.0652	183.0654	0.20	123.0439，155.0703，95.0489	C_9_H_10_O_4_	Syringaldehyde	[M + H]^+^
79	4.71	163.0390	163.0390	0.55	135.0442，105.0447，95.0492	C_9_H_6_O_3_	7-Hydroxycoumarin	[M + H]^+^
80	4.82	295.1904	295.1890	−4.69	277.1191，117.1025	C_17_H_26_O_4_	6-Gingerol	[M + H]^+^
81	5.30	181.0718	181.0723	2.76	163.0615，101.0245，89.0244，71.0139，59.0138	C_6_H_14_O_6_	Mannitol	[M-H]^−^

Methodological rationale: Preliminary solvent screening (ethanol, water, ethyl acetate, n-butanol, petroleum ether) revealed superior pharmacological activity in aqueous extracts, thus justifying aqueous extraction. Dose-response evaluation of three concentrations (1, 2.12, and 4 g/mL) established 2.12 g/mL as the optimal extraction concentration for subsequent analyses.

#### 3.1.1 Structure analysis of glucosinolates

Glucosinolates constitute the principal phytochemical constituents in MOS. Structurally classified by their side chain characteristics, these compounds are categorized into three groups: (1) aliphatic glucosinolates with linear/branched alkyl chains; (2) aromatic glucosinolates containing benzene rings; and (3) indolic glucosinolates featuring indole ring systems (Ares, Valverde, Nozal, Bernal, & Bernal, 2016). Our analysis identified five glucosinolate derivatives in M. oleiferaseeds, predominantly aromatic subtypes. To elucidate their fragmentation mechanisms, glucomoringin was selected as a representative compound for detailed mass spectrometric characterization.

**Fig. 2:**
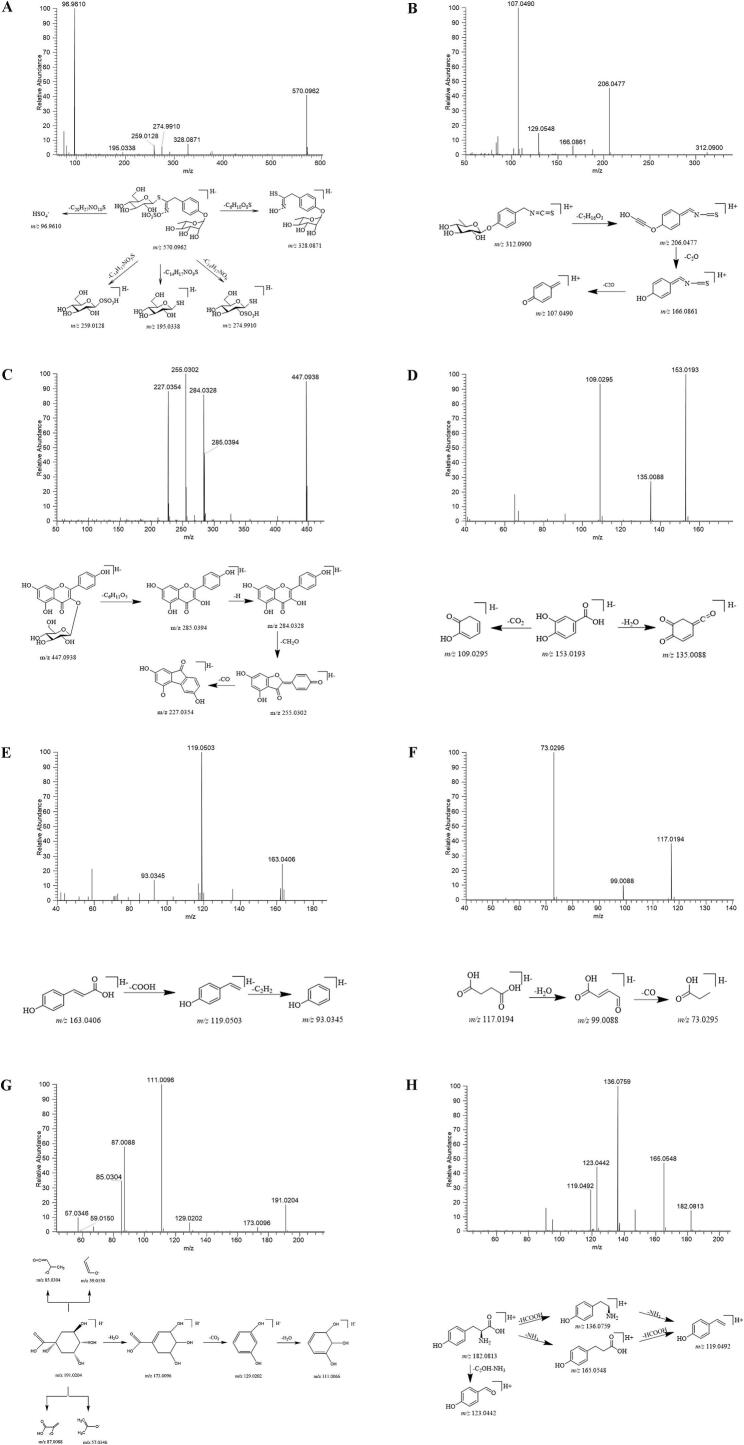
Structure resolution of the major compounds in MOS. (A) Fragmentation pattern of Glucomoringin. (B) Fragmentation pattern of moringin. (C) Fragmentation pattern of Astragalin. (D) Fragmentation pattern of Protocatechuic acid. (E) Fragmentation pattern of p-coumaric acid. (F) Fragmentation pattern of Succinic acid. (G) Fragmentation pattern of Quinic acid. (H) Fragmentation pattern of L-Tyrosine.

In negative ionization mode, the precursor ion ([M-H]^−^ at *m*/*z* 570.0962) exhibited three distinct fragmentation pathways (Fig. 2A). Pathway 1: Direct neutral loss of HSO_4_^−^ (97.9674 Da), yielding the diagnostic fragment at m/z 96.9610. Pathway 2: Cleavage of the C (1′)-S bond followed by sequential loss of the glucose moiety (162.0528 Da) and aromatic substituents, generating characteristic fragments at m/z 328.0871 and 74.9910. Pathway 3: S—C bond scission adjacent to the thioglucose unit, producing the m/z 195.0338 fragment. Subsequent sulfate-hydroxyl exchange at the C(2) position of glucose yielded complementary ions at m/z 259.0128 and 274.9910, consistent with established fragmentation patterns (Jaja-Chimedza et al., 2018; Prieto, López, & Simal-Gandara, 2019).

#### 3.1.2 Structure analysis of alkaloid compounds

Alkaloids represent a structurally diverse class of nitrogen-containing secondary metabolites characterized by basic heterocyclic frameworks. These compounds are systematically categorized based on their core C—N skeletal architectures, encompassing subclasses such as pyrrolidines, pyridine derivatives, quinolines, isoquinolines, and indole alkaloids. Phytochemical profiling of MOS has revealed seven phenethylamine-type alkaloids, three of which—moringin, marumoside A, and niazinin A—are recognized as chemotaxonomic markers unique to this species (Abdelsayed, Abdel Motaal, & Hanafi, 2021; Gao et al., 2022; Ma et al., 2018). These compounds share conserved structural motifs comprising a β-d-glucopyranosyl unit linked to a phenethylamine core via O-glycosidic bonding.

The fragmentation behavior is exemplified by moringin ([M + H]^+^ *m*/*z* 312.0900) under positive ionization (Fig. 2B). Primary cleavage occurs at the glycosidic bond (C—O, m/z − 146.0423), generating aglycone fragments at m/z 166.0865 (phenethylamine moiety) and m/z 206.0477 (glucosyl‑oxygenated intermediate). Subsequent isothiocyanate elimination (−97 Da, C₂H₃NS) induces aromatic ring rearrangement, yielding the diagnostic ion at m/z 107.0490 (C₆H₅NO^−^), indicative of ortho-aminophenolic structural reorganization.

#### 3.1.3 Structure analysis of flavonoids and flavonoid glycosides

Flavonoids constitute a class of polyphenolic compounds characterized by a 15‑carbon skeleton comprising two aromatic rings (A and B) interconnected via a heterocyclic pyran ring (C-ring). These phytochemicals are systematically classified into subclasses—including flavonols, flavones, isoflavones, dihydroflavones, and dihydroisoflavones—based on structural variations in the C-ring configuration, inter-ring conjugation patterns, and oxidation states of the central carbon framework. During mass spectrometric analysis, flavonoids undergo characteristic fragmentation patterns in both ionization modes, predominantly through cleavage of the C-ring followed by sequential neutral losses (CO: 28 Da, CO₂: 44 Da, H₂O: 18 Da). The resultant A-ring and B-ring-derived fragments serve as diagnostic markers for structural elucidation.

Five distinct flavonoid subclasses have been identified in MOS through advanced analytical methodologies (Adebayo, Arsad, & Samian, 2018; Liao, Cheng, Diao, Sun, & Zhang, 2017; Zhou et al., 2022). To illustrate the fragmentation behavior, astragalin (*m*/*z* 447.0938 [M-H]^−^) demonstrates a representative cleavage pathway (Fig. 2C) (Zhou et al., 2022). Initial glycosidic bond rupture generates fragm227.0354 cross 285.0394 (aglycone) and *m*/*z* 284.0328 (radical aglycone). Subsequent C-ring-centered fragmentation yields characteristic daughter ions at *m*/*z* 255.0302 (retro-Diels-Alder product) and m/z 227.0354 (cross-ring cleavage derivative), confirming the compound's substitution pattern.

#### 3.1.4 Structure analysis of phenolic acids

Phenolic acids represent a class of aromatic phytochemicals characterized by hydroxyl and carboxyl substituents on their benzene nuclei, conferring strong ionization efficiency in negative ion mode during mass spectrometric analysis. Based on their carbon frameworks, these compounds are subclassified into two structural archetypes: (1) C_6_-C_1_ derivatives (benzoic acid derivatives, e.g., protocatechuic acid) and (2) C_6_-C_3_ derivatives (cinnamic acid derivatives, e.g., p-coumaric acid). The characteristic fragmentation patterns of these subclasses are exemplified through protocatechuic acid and p-coumaric acid analyses.

The deprotonated molecular ion [M-H]^−^ of protocatechuic acid (*m*/*z* 153.0193) undergoes sequential decarboxylation (−44 Da, CO_2_ loss) followed by dehydration (−18 Da, H_2_O loss), generating diagnostic fragment ions at *m*/*z* 109.0292 and m/z 135.0088, respectively (Fig. 2D). This cleavage pattern aligns with established fragmentation pathways for ortho-dihydroxybenzoic acid derivatives (Zhou et al., 2022). Similarly, p-coumaric acid ([M-H]^−^ *m*/*z* 163.0406) demonstrates a primary neutral loss of CO_2_ (44 Da) yielding *m*/*z* 119.0503, with subsequent side-chain cleavage producing the *m*/*z* 93.0345 fragment through allylic bond rupture (Fig. 2E).

#### 3.1.5 Structure analysis of organic acid compounds

Organic acids constitute a class of phytochemicals characterized by the presence of at least one carboxyl functional group (-COOH) in their molecular architecture, exhibiting optimal ionization efficiency in negative ion mode during mass spectrometric analysis. Their fragmentation patterns are dominated by characteristic decarboxylation (−44 Da, CO_2_ loss). High-resolution tandem mass spectrometry has enabled the identification of eight distinct organic acid derivatives in MOS.

Under negative ionization conditions, succinic acid ([M-H]^−^ *m*/*z* 117.0194) demonstrates sequential neutral losses, yielding diagnostic fragment ions at *m*/*z* 99.0088 (−18 Da, H₂O loss) and m/z 73.0295 (additional CO₂ elimination) (Fig. 2F). Similarly, quinic acid ([M-H]^−^ *m*/*z* 191.0204) exhibits two predominant fragmentation pathways. A cascade of dehydration (−18 Da) → decarboxylation (−44 Da) → secondary dehydration (−18 Da), generating successive fragments at *m*/*z* 173.0096 → m/z 129.0202 → m/z 111.0096; Six-membered ring cleavage via retro-aldol fragmentation, producing complementary ions at m/z 87.0088 (C₃H₃O₃^−^) with its dehydrated form m/z 85.0304, and m/z 57.0346 (C₂H₅O₂^−^) accompanied by m/z 59.0150 (C₂H₃O₂^−^) (Fig. 2G).

#### 3.1.6 Structure analysis of amino acid compounds

Amino acids constitute essential nutritional components in biological systems. Tandem mass spectrometric analysis has identified eight distinct amino acid derivatives in MOS, corroborating previous phytochemical investigations ((Piraud et al., 2003; Zhou et al., 2022). The fragmentation behavior of these compounds is exemplified through the analysis of L-tyrosine (Fig. 2H). The protonated molecular ion [M + H]^+^ of L-tyrosine (m/z 182.0813) undergoes two principal cleavage pathways. Intramolecular rearrangement yielding the m/z 123.0442 fragment via neutral loss of NH₃ (−17 Da) and CO₂ (−44 Da); Sequential decarboxylation (−44 Da) followed by deamination (−17 Da), generating successive fragments at m/z 165.0548 → m/z 136.0759 → m/z 119.0492 through α‑carbon backbone cleavage.

Additional phytoconstituents identified in MOS extracts include polyphenolic derivatives (e.g., 6-gingerol) and aromatic aldehydes (protocatechualdehyde, vanillin), with complete structural assignments and spectral data presented in Table 1.

### 3.2 Results of identification of transitional components in serum

TIC of blank serum and drug-containing serum were acquired in both positive and negative ionization modes. Using Xcalibur 4.1 data processing software, background interference was subtracted from post-administration sample chromatograms, yielding processed TIC with blank subtraction as illustrated in Supplementary Figs. 1 and 2. Subsequent analysis with TraceFinder 4.1 software identified 11 prototype constituents in serum samples, comprising 2 glucosinolates, 2 alkaloids, 1 flavonoid, 2 phenolic acids, and 4 miscellaneous compounds, with detailed characterization data presented in Table 2. All detected components demonstrated mass spectral consistency with reference MOS standards in vitro.

**Table 2:** Identification of the prototype components from compound MOS granule in serum of rats.

No.	TR	Theoretical *m/z* (Da)	Measured m/z(Da)	Error (ppm)	MS/MS	Predicted Formula	Compounds	Type
P1	1.27	137.0244	137.0245	−0.73	137.0244,135.9714,109.1268	C_7_H_6_O_3_	Protocatechualdehyde	[M-H]^−^
P2	1.72	163.0400	163.0406	−3.68	162.8394,119.0503	C_9_H_8_O_3_	p-Coumaric acid	[M-H]^−^
P3	3.30	408.0428	408.0431	−0.74	96.960,80.9653	C_14_H_19_NO_9_S_2_	Glucotropaeolin	[M-H]^−^
P4	3.33	570.0962	570.0957	0.88	96.960,80.965,74.9910,328.0859,259.0128,274.9902	C_2_0H_2_9NO_1_4S_2_	Glucomoringin	[M-H]^−^
P5	3.37	328.1391	328.1398	−2.13	292.1184,264.1235,166.0865,97.0285,69.0337	C_15_H_21_NO_7_	methyl N-[[4-(3,4,5-trihydroxy-6-methyloxan-2-yl)oxyphenyl]methyl]carbamate isomer 2	[M + H]^+^
P6	3.61	312.0900	312.0912	−3.85	107.0490,166.0865,79.0541	C_14_H_17_NO_5_S	Moringin	[M + H]^+^
P7	3.87	153.0192	153.0194	−1.05	109.0295,135.0086	C_7_H_6_O_4_	Protocatechuic acid	[M-H]^−^
P8	3.97	151.0402	151.0400	1.66	92.027,136.0168	C_8_H_8_O_3_	Vanillin	[M-H]^−^
P9	4.01	433.1124	433.1117	1.62	283.0615,313.0718,271.0593,163.0596,85.0282	C_21_H_20_O_10_	Vitexin	[M + H]^+^
P10	4.69	183.0652	183.0663	4.10	123.0439,155.0703,95.049	C_9_H_10_O_4_	Syringaldehyde	[M + H]^+^
P11	5.03	293.1759	293.1762	−1.02	236.11,221.1548,177.0922	C_17_H_26_O_4_	6-Gingerol	[M-H]^−^

Compound Discoverer (CD) software was employed for structural elucidation through systematic analysis of mass-to-charge ratios, isotopic distribution patterns, and characteristic fragmentation profiles. Comparative analysis of molecular ions and secondary fragment ions against established metabolite databases and published literature enabled tentative identification of metabolic derivatives. This comprehensive approach revealed 27 distinct MOS-derived metabolites in rat serum following oral administration, with complete analytical results tabulated in Table 3.

**Table 3:** Identification of the metabolites from compound MOS granule in serum of rats.

No.	TR	Theoretical *m/z* (Da)	Measured m/z(Da)	Error (ppm)	MS/MS	Predicted Formula	Compounds	Type
M1	1.08	216.9812	216.9816	−1.54	93.035,137.0246,172.9930	C_7_H_6_O_6_S	Protocatechualdehyde Sulfation	[M-H]^−^
M2	1.59	194.0459	194.0453	3.09	150.0561,93.035	C_9_H_9_NO_4_	Protocatechualdehyde Glycine	[M-H]^−^
M3	1.68	344.1340	344.1326	4.06	308.1130,280.1082,182.08136,137.0793,97.029,69.0337	C_15_H_21_NO_8_	methyl N-[[4-(3,4,5-trihydroxy-6-methyloxan-2-yl)oxyphenyl]methyl]carbamate isomer 2 Hydroxylation	[M + H]^+^
M4	1.79	326.1057	326.1047	3.07	92.027,206.0477,136.0758	C_15_H_19_NO_5_S	Moringin Methylation	[M + H]^+^
M5	2.40	208.0615	208.0616	−0.55	134.0613,149.0482	C_10_H_11_NO_4_	Vanillin Glycine	[M-H]^−^
M6	3.40	385.1605	385.1615	−2.62	343.1160,133.0612,107.04939	C_17_H_24_N_2_O_8_	methyl N-[[4-(3,4,5-trihydroxy-6-methyloxan-2-yl)oxyphenyl]methyl]carbamate isomer 2 Glycine	[M + H]^+^
M7	3.52	232.9761	232.9752	3.86	109.0290,135.0435	C_7_H_6_O_7_S	Protocatechuic acid Sulfation	[M-H]^−^
M8	3.63	166.0321	166.0325	2.22	107.0493,120.0809	C_8_H_7_NOS	4-Hydroxybenzyl isothiocyanate	[M + H]^+^
M9	3.64	458.1992	4458.1955	2.44	107.0493,206.0598,	C_19_H_27_N_3_O_8_S	Moringin Glutamine conjugation	[M + H]^+^
M10	3.65	633.2072	633.2046	4.12	253.10696,107.0493	C_25_H_36_N_4_O_13_S	methyl N-[[4-(3,4,5-trihydroxy-6-methyloxan-2-yl)oxyphenyl]methyl]carbamate isomer 2 GSHconjugation	[M + H]^+^
M11	3.67	242.9968	242.9961	2.78	163.0406,119.0505	C_9_H_8_O_6_S	p-Coumaric acid Sulfation	[M-H]^−^
M12	3.74	388.1602	388.1606	−0.91	301.1299,203.0530,165.0550,133.0550,130.0500	C_17_H_25_NO_9_	methyl N-[[4-(3,4,5-trihydroxy-6-methyloxan-2-yl)oxyphenyl]methyl]carbamate isomer 2 Hydration Acetylation	[M + H]^+^
M13	3.79	386.1142	386.1149	−1.82	107.0492,89.0598,241.0647	C_16_H_21_N_2_O_7_S	Moringin Glycine	[M + H]^+^
M14	3.92	354.1006	354.0991	4.25	107.04922,217.08557,129.0544	C_16_H_19_NO_6_S	Moringin Acetylation	[M + H]^+^
M15	4.17	463.0882	463.0870	2.51	287.0930,175.0527	C_21_H_20_O_12_	Vitexin Hydroxylation	[M-H]^−^
M16	4.19	463.1237	463.1235	0.54	283.1695,337.1633	C_22_H_22_O_11_	Vitexin Hydroxylation Methylation	[M + H]^+^
M17	4.53	528.0548	528.0532	−0.82	80.9652,96.960	C_17_H_25_N_2_O_11_S_3_	Glucotropaeolin Cysteine	[M-H]^−^
M18	4.59	291.1602	291.1599	1.03	221.1547,179.0718,	C_17_H_24_O_4_	6-Gingerol Dehydrogenation	[M-H]^−^
M19	4.63	177.0557	177.0559	−1.08	162.0322,145.0297,119.0462	C_10_H_10_O_3_	p-Coumaric acid Methylation	[M-H]^−^
M20	4.67	240.0866	240.0824	1.75	95.086,123.1170,183.1746	C_14_H_16_N_2_O_5_	Syringaldehyde Glycine	[M + H]^+^
M21	4.68	584.0749	584.0752	−0.52	96.9610,424.0403,328.0854,259.0140, 195.0805	C_20_H_27_NO_15_S_2_	Glucotropaeolin glucuronide	[M-H]^−^
M22	4.82	277.1798	277.1806	−2.78	137.0594,277.1181,179.1063	C_17_H_24_O_3_	6-Gingerol Desaturation	[M + H]^+^
M23	4.89	275.1642	275.1636	1.93	275.1646,159.0807	C_17_H_22_O_3_	6-Gingerol Desaturation Dehydrogenation	[M + H]^+^
M24	5.02	307.1915	307.1911	1.32	293.2226,235.1326	C_18_H_28_O_4_	6-Gingerol Methylation	[M + H]^+^
M25	5.18	352.2129	352.2137	−2.27	177.0929,277.1796	C_19_H_31_NO_5_	6-Gingerol Glycine	[M-H]^−^
M26	5.29	281.2475	281.2461	4.97	147.0653,135.0806,107.0856	C_18_H_32_O_2_	6-Gingerol Reduction Methylation	[M + H]^+^
M27	7.99	607.1304	593.1517	1.20	431.3503,311.2234,283.2651,413.8943	C_27_H_30_O_15_	Vitexin Glucuronide	[M-H]^−^

#### 3.2.1 Identification of glucosinolates metabolites

Comparative analysis between authentic and blank serum samples using the aforementioned data processing methodology enabled the preliminary identification of two glucosinolate prototype constituents and two metabolic derivatives. Metabolic profiling revealed that glucosinolates primarily undergo phase I hydrolytic metabolism (deglycosylation) followed by phase II conjugation reactions, including glucuronidation and cysteinyl conjugation.

**Fig. 3:**
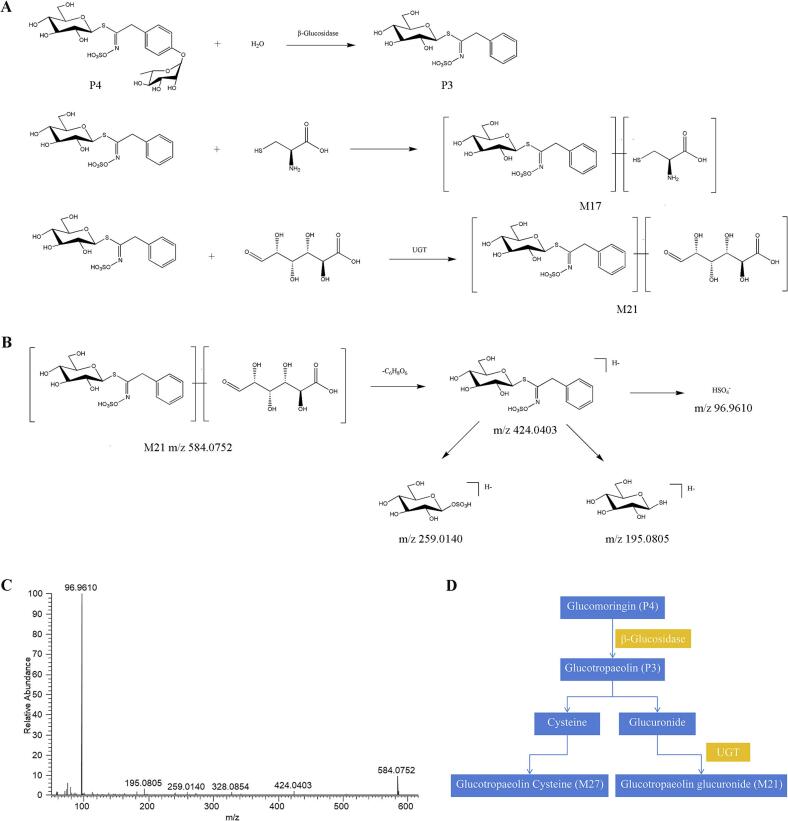
Analysis of prototype components and metabolite lysis pathway of glucosinolate in vivo. (A) The possible metabolic pathways of glucomoringin in the rat serum. (B) Proposed fragmentation pathways of M21 in the negative ion mode. (C) Product ion spectra of M21 in the negative ion mode. (D) The possible interactive metabolic network diagram of glucomoringin in the rat serum.

The metabolic interconversion network between glucomoringin (P4) and its derivatives in serum is illustrated in Fig. 3A and D. Hydrolytic removal of a glucose residue from glucomoringin yields glucotropaeolin (P3), which subsequently undergoes phase II modifications via glucuronyl and cysteinyl conjugation to produce secondary metabolites M17 and M21, respectively. Representative metabolite M21 was selected for fragmentation pathway elucidation. In negative ionization mode, M21 exhibited a deprotonated molecular ion ([M-H]^−^ at *m*/*z* 584.0752), corresponding to the molecular formula C_20_H_27_NO_15_S_2_ as calculated by CD software. Collision induced dissociation (CID) generated characteristic fragment ions at m/z 424.0403, 328.0854, 259.0140, and 96.9610, demonstrating fragmentation patterns consistent with glucotropaeolin-derived conjugates. While spectral data confirmed conjugation with cysteine, the specific binding site remained undetermined. Based on these analytical findings, M21 was tentatively characterized as a cysteinyl-conjugated glucotropaeolin derivative. The complete mass spectral profile and proposed fragmentation pathway are presented in Fig. 3B and C.

#### 3.2.2 Identification of alkaloid metabolites

**Fig. 4:**
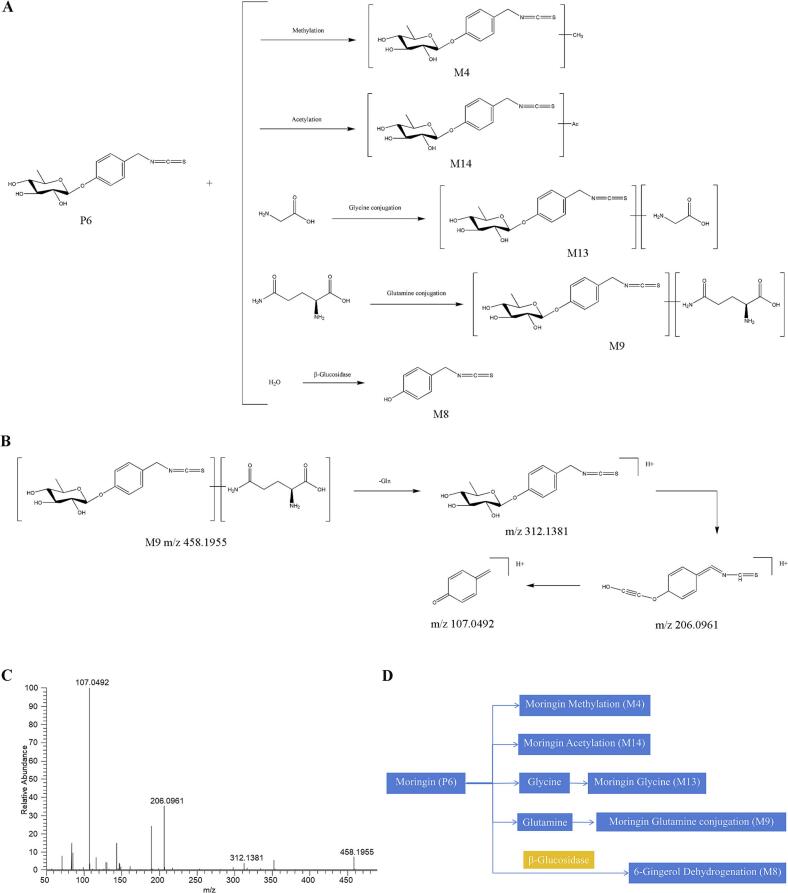
Analysis of prototype components and metabolite lysis pathway of alkaloid in vivo. (A) The possible metabolic pathways of moringin in the rat serum. (B) Proposed fragmentation pathways of M9 in the positive ion mode. (C) Product ion spectra of M9 in the positive ion mode. (D) The possible interactive metabolic network diagram of moringin in the rat serum.

Serum analysis of rats revealed two alkaloid prototype constituents and nine metabolic derivatives. Metabolic profiling indicated predominant phase II conjugation reactions including acetylation, methylation, glucuronidation, and glutamine conjugation (Fig. 4A and D). Using moringin (P6) as a representative compound, we characterized its biotransformation pathway involving phase II modifications to generate acetylated (M14), methylated (M4), glycine-conjugated (M13), and glutamine-conjugated (M9) derivatives, alongside phase I hydrolytic deglycosylation yielding 4-hydroxybenzyl isothiocyanate (M8).

For metabolite M9, structural elucidation was performed via mass spectrometric analysis. In positive ionization mode, M9 exhibited a protonated molecular ion ([M + H]^+^ at *m*/*z* 458.1955), corresponding to the molecular formula C_19_H_27_N_3_O_8_S as calculated by CD software. CID generated diagnostic fragment ions at *m*/*z* 312.1381, 206.0961, and 107.0492, exhibiting high concordance with characteristic moringin-derived fragments. While spectral patterns confirmed glutamine conjugation, the specific binding site remained undetermined. Based on these findings, M13 was tentatively identified as a moringin-glutamine conjugate. The complete mass spectral data and proposed fragmentation pathway are illustrated in Fig. 4B and C.

#### 3.2.3 Identification of flavonoids metabolites

**Fig. 5:**
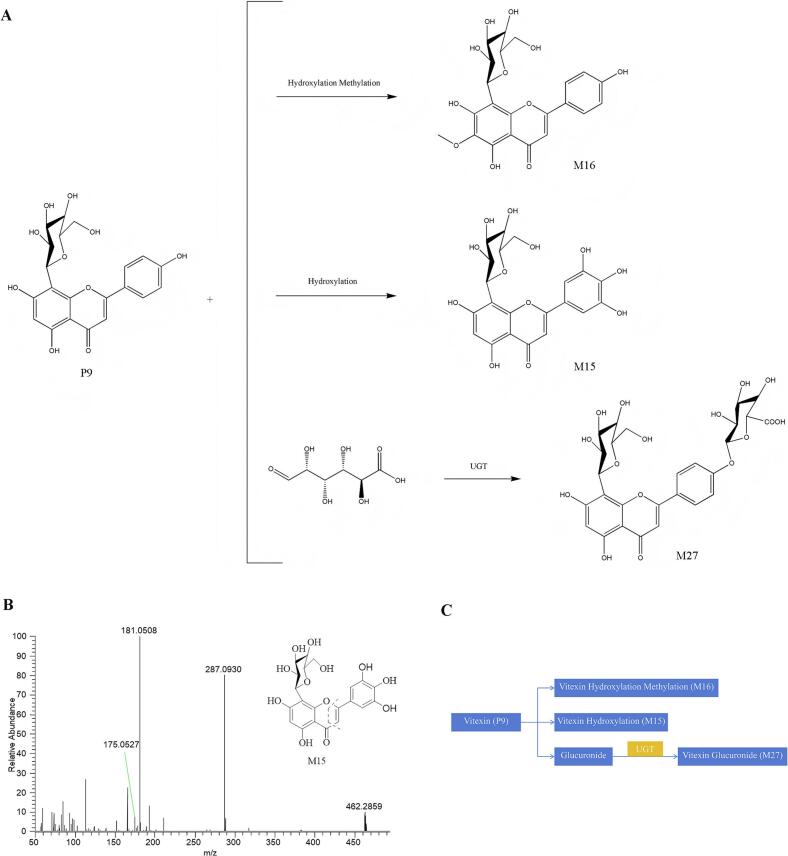
Analysis of prototype components and metabolite lysis pathway of flavonoid in vivo. (A) The possible metabolic pathways of flavonoids in the rat serum. (B) Product ion spectra of M15 in the positive ion mode. (C) The possible interactive metabolic network diagram of vitexin in the rat serum.

Serum analysis following intragastric administration of MOS aqueous extract revealed four flavonoid derivatives, comprising one prototype constituent and three metabolic derivatives (Najmanová, Vopršalová, Saso, & Mladěnka, 2020; Yue et al., 2022), The proposed biotransformation pathway for these compounds is depicted in Fig. 5A and C. Representative metabolite M15 was selected to demonstrate its fragmentation behavior. The metabolite M15 was taken as an example to illustrate the cleavage pathway of this compound.

In positive ionization mode, M15 displayed a protonated molecular ion ([M + H]^+^ at *m*/*z* 463.0870), corresponding to the molecular formula C_21_H_20_O_12_ as calculated via CD software. CID generated characteristic fragment ions at m/z 287.0930 and 175.0527, consistent with retro-Diels-Alder fragmentation of the flavonoid C-ring. Structural analysis suggested hydroxylation modifications on the B-ring of the flavonoid aglycone, though the specific substitution site remained undetermined. Based on these spectral features, M15 was tentatively identified as a polyhydroxylated vitexin derivative. The full mass spectral profile and proposed fragmentation pathway are illustrated in Fig. 5B.

#### 3.2.4 Identification of phenolic acids metabolites

**Fig. 6:**
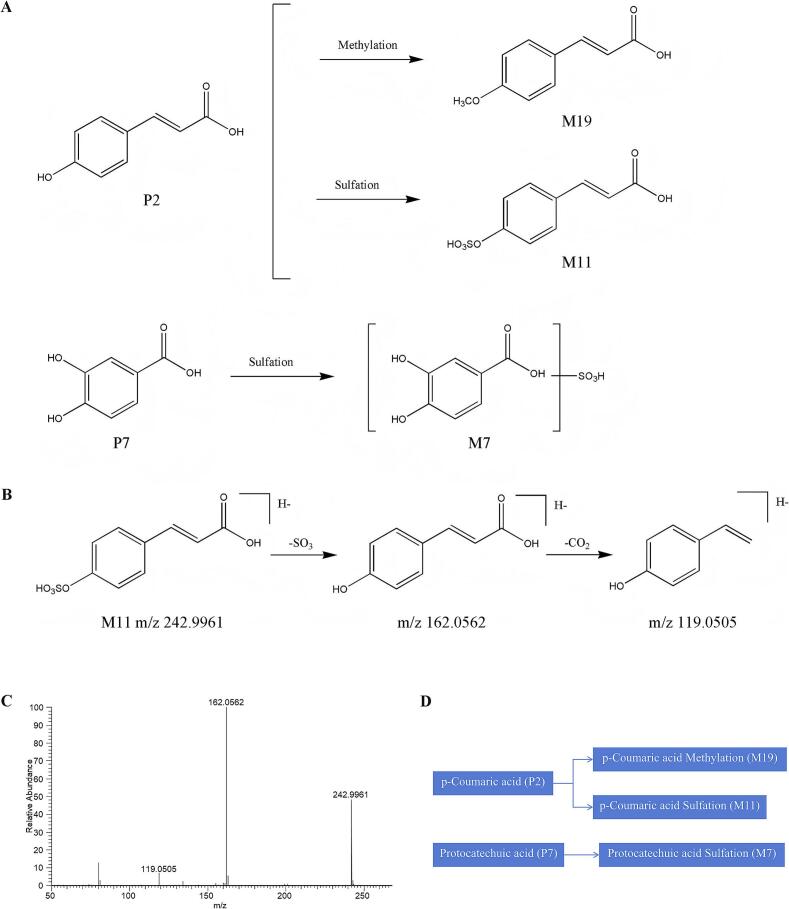
Analysis of prototype components and metabolite lysis pathway of phenolic acids in vivo. (A) The possible metabolic pathways of phenolic acids in the rat serum. (B) Proposed fragmentation pathways of M11 in the negative ion mode. (C) Product ion spectra of M11 in the negative ion mode. (D) The possible interactive metabolic network diagram of phenolic acids in the rat serum.

Comparative analysis with blank biological matrices identified five phenolic acid derivatives in serum samples from rats receiving MOS aqueous extract via intragastric administration, comprising two prototype constituents and three metabolic derivatives. The biotransformation processes predominantly involved methylation and sulfation conjugation, with the proposed metabolic pathway illustrated in Fig. 6A and D.

Representative metabolite M11 was selected to elucidate its fragmentation pathway. In negative ionization mode, M11 exhibited a precursor ion ([M-H]^−^ at *m*/*z* 242.0132), corresponding to the molecular formula C₉H₈O₆S as calculated by CD software. CID generated characteristic secondary fragment ions at m/z 162.0562 and 119.0505, consistent with the diagnostic fragmentation pattern of p-coumaric acid derivatives. These spectral features supported the structural assignment of M11 as a p-coumaric acid-sulfate conjugate. The full mass spectral profile and proposed fragmentation pathway are presented in Fig. 6B and C.

#### 3.2.5 Identification of other metabolite

**Fig. 7:**
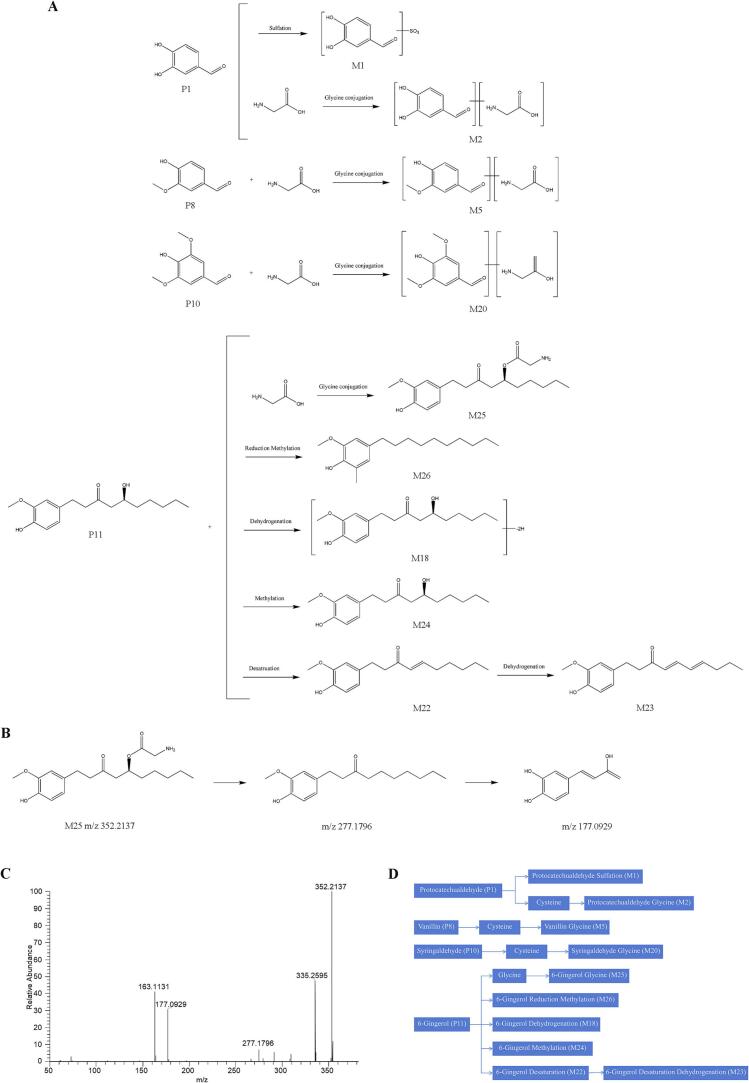
Analysis of prototype components and metabolite lysis pathway of others in vivo. (A) The possible metabolic pathways of others in the rat serum. (B) Proposed fragmentation pathways of M25 in the negative ion mode. (C) Product ion spectra of M25 in the negative ion mode. (D) The possible interactive metabolic network diagram of others in the rat serum.

Pharmacokinetic analysis revealed 14 additional compounds in post-administration rat serum, comprising 4 prototype constituents and 10 metabolic derivatives (S. Z. Jiang, Wang, & Mi, 2008). The prototype constituents consisted of three aldehyde derivatives and one polyphenolic compound. Aldehyde metabolism primarily involved phase II conjugation reactions, with the postulated biotransformation pathway illustrated in Fig. 7A and C. Polyphenolic compounds underwent phase I dehydrogenation followed by phase II modifications including methylation and glycine conjugation, as detailed in Table 2, Table 3. Representative metabolite M25 ([M-H]^−^ with m/z 352.2137) demonstrated characteristic fragmentation patterns through collision-induced dissociation, generating diagnostic fragment ions at m/z 177.0929 and 277.1796 corresponding to 6-gingerol derivatives. Structural elucidation confirmed M26 as a 6-gingerol-glycine conjugate, with its secondary fragmentation patterns and proposed cleavage pathway presented in Fig. 7B and D.

### 3.3 Pharmacokinetic results

#### 3.3.1 LC-MS/MS method validation

##### 3.3.1.1 Selectivity

Method selectivity was confirmed by comparative chromatographic analysis of blank plasma, fortified plasma, and post-administration samples (Supplementary Fig. 3). No endogenous or metabolic interference was observed at retention times corresponding to 6-gingerol, vanillin, eugenol, or IS.

##### 3.3.1.2 Calibration curve and LLOQ

Linear ranges were established: 3.57–1785 ng/mL (6-gingerol), 1.772–886 ng/mL (vanillin), and 4.056–2028 ng/mL (syringaldehyde). All analytes demonstrated satisfactory linearity (*r* > 0.99) within their respective ranges. Calibration parameters and lower quantification limits (LLOQ) are detailed in Supplementary Table 1.

##### 3.3.1.3 Precision and accuracy

Intra-day and inter-day precision (RSD ≤ 15 %) and accuracy (RE within ±15 %) met bioanalytical validation criteria. Results across QC levels (*n* = 18) complied with acceptance limits (Supplementary Table 2).

##### 3.3.1.4 Extraction recovery and matrix effect

Mean extraction recoveries exceeded 80.9 % for all analytes (IS recovery: 90.8 %). The internal standard-normalized matrix factor exhibited RSD < 15 % across six plasma lots (Supplementary Table 3).

##### 3.3.1.5 Stability

Analytes remained stable (RSD < 10 %) under all tested conditions: ambient temperature (24 h), post-preparative (12 h, 4 °C), freeze-thaw cycles (3×), and long-term storage (−80 °C, 15 d) (Supplementary Table 4).

#### 3.3.2 Pharmacokinetic studies

**Fig. 8:**
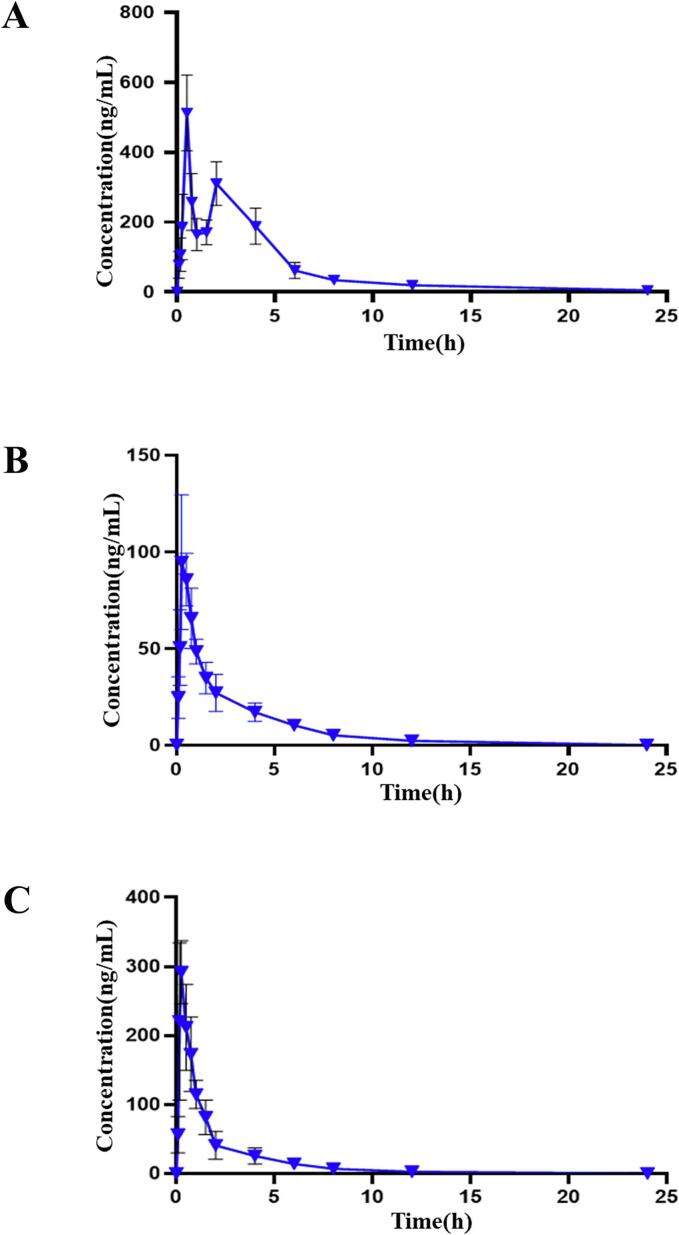
Mean plasma concentration-time curves of each component in the administered rats. (A) Mean plasma concentration-time profiles of 6-Gingerol. (B) Mean plasma concentration-time profiles of Vanillin. (C) Mean plasma concentration-time profiles of Syringaldehyde (mean ± SD, *n* = 6).

LC-MS/MS quantified plasma concentrations of three bioactive components following oral administration of MOS aqueous extract in rats. Mean plasma concentration-time profiles (Fig. 8) and pharmacokinetic parameters (Supplementary Table 5) were established.

The validated method simultaneously determined 6-gingerol, vanillin, and syringaldehyde concentrations across timepoints. Chromatographic optimization revealed superior analyte response in negative ionization mode using methanol-0.1 % aqueous formic acid versus acetonitrile-water or methanol-water systems, yielding enhanced ionization efficiency and reduced background noise. Plasma pretreatment methodologies were evaluated, with protein precipitation demonstrating comparable extraction efficiency to liquid-liquid extraction (chloroform/ethyl acetate/isopropanol) while offering operational advantages.

6-Gingerol exhibited biphasic absorption kinetics, potentially attributable to enterohepatic recirculation (Wang et al., 2014) and metabolic interconversion between parent compound and glucuronide metabolites (Feng et al., 2018). The initial absorption peak (T₁ ≈ 0.5 h) corresponds to direct intestinal absorption, while the secondary peak (T₂ ≈ 4–6 h) likely reflects microbial deglucuronidation and subsequent reabsorption. Vanillin and syringaldehyde demonstrated rapid pharmacokinetics: T_max_: 0.33 ± 0.13 h (vanillin); 0.28 ± 0.11 h (syringaldehyde). t_1/2_: 2.54 ± 0.82 h (vanillin); 3.34 ± 2.30 h (syringaldehyde).

These parameters indicate swift distribution and elimination, consistent with the steep terminal elimination phases observed in Fig. 8. The established pharmacokinetic profiles provide critical insights for clinical translation of MOS-based formulations.

## 4 Conclusion

This study systematically characterized MOS constituents in vitro and in vivo using UPLC-Q-Exactive Orbitrap-MS. We identified 81 chemical components in aqueous extracts and 38 blood-borne compounds (11 prototypes; 27 metabolites) in rat serum, establishing diagnostic fragmentation patterns for key phytochemicals. The developed UPLC-MS/MS method enabled rapid quantification of 6-gingerol, vanillin, and syringaldehyde pharmacokinetics in rat plasma, revealing biphasic absorption kinetics for 6-gingerol and rapid elimination profiles for all three markers.

Translational implications: Sulfated metabolites (e.g., M6, M7 in Supplementary Data) identified during in vivo analysis may serve as stability markers for MOS-based functional foods, given their resistance to enzymatic degradation. The quantified phenolic compounds (vanillin, syringaldehyde) could be potentially applied as standardization markers for nutraceutical formulations targeting metabolic disorders.

Study limitations and future directions: While enterohepatic recirculation was implicated in 6-gingerol pharmacokinetics, fecal sampling was not performed, potentially overlooking gut microbiota-mediated metabolites like deglycosylated flavonoids. Future investigations should integrate metabolomic profiling of intestinal contents to comprehensively map microbial biotransformation pathways.

These findings provide both a chemical foundation for MOS quality control and pharmacokinetic insights guiding clinical translation of MOS-derived therapeutics.

## CRediT authorship contribution statement

**Jiahong Wang:** Writing – original draft, Methodology, Funding acquisition, Formal analysis, Conceptualization. **Juan Cao:** Validation, Methodology, Investigation. **Hao Wang:** Validation, Methodology, Data curation. **Yudie Zhang:** Validation, Software, Methodology. **Li Jiang:** Visualization, Validation. **Jiaohan Zhan:** Validation, Methodology. **Yanxiu Sun:** Visualization, Validation, Software. **Yiyang Du:** Methodology, Funding acquisition, Conceptualization. **Tingxu Yan:** Methodology, Funding acquisition, Formal analysis. **Ying Jia:** Resources, Project administration, Funding acquisition. **Bosai He:** Writing – review & editing, Supervision, Conceptualization.

## Declaration of competing interest

The authors declare that they have no known competing financial interests or personal relationships that could have appeared to influence the work reported in this paper.

## Data Availability

The raw data supporting this study have been deposited in the OMIX repository at China National Center for Bioinformation (CNCB/NGDC) under accession number [OMIX011128]. Public link: https://ngdc.cncb.ac.cn/omix/repository/OMIX011128.

## Appendix A Supplementary data

Supplementary material Image 1
