# Supplementary material for: In vitro identification and in vivo metabolic profiling of chemical constituents in Moringa oleifera seeds
Source: Food Chem X. 2025 Aug 8;30:102899. doi: 10.1016/j.fochx.2025.102899 (PMC12391776; doi:10.1016/j.fochx.2025.102899)
Supplement: Supplementary file 1 — Supplementary material [file mmc1.docx]

**Supplementary data**

**In Vitro Identification and In Vivo Metabolic Profiling of Chemical Constituents in Moringa oleifera Seeds**

Jiahong Wang^b^, Juan Cao^c^, Hao Wang^b^, Yudie Zhang^b^, Li Jiang^c^, Jiaohan Zhan^c^, Yanxiu Sun^c^, Yiyang Du^a^, Tingxu Yan^a^, Ying Jia^a*^, Bosai He^a*^

^a^School of Functional Food and Wine, Shenyang Pharmaceutical University, Wenhua Road 103, Shenyang 110016, China

^b^ School of Life Science and Biopharmaceutics, Shenyang Pharmaceutical University, Shenyang 110016, China

^c^ School of Pharmacy, Shenyang Pharmaceutical University, Wenhua Road 103, Shenyang 110016, China

* Corresponding authors.

1. Bosai He

Address: School of Functional Food and Wine, Shenyang Pharmaceutical University, Wenhua Road 103, Shenyang 110016, China

Email：hbspharma@163.com

Phone：+86-13604075835

Fax：+86-24-23986189

2. Ying Jia

Address: School of Functional Food and Wine, Shenyang Pharmaceutical University, Wenhua Road 103, Shenyang 110016, China

Email：jiayingsyphu@126.com

Phone：+86-18640055196

Fax：+86-24-23986189

**
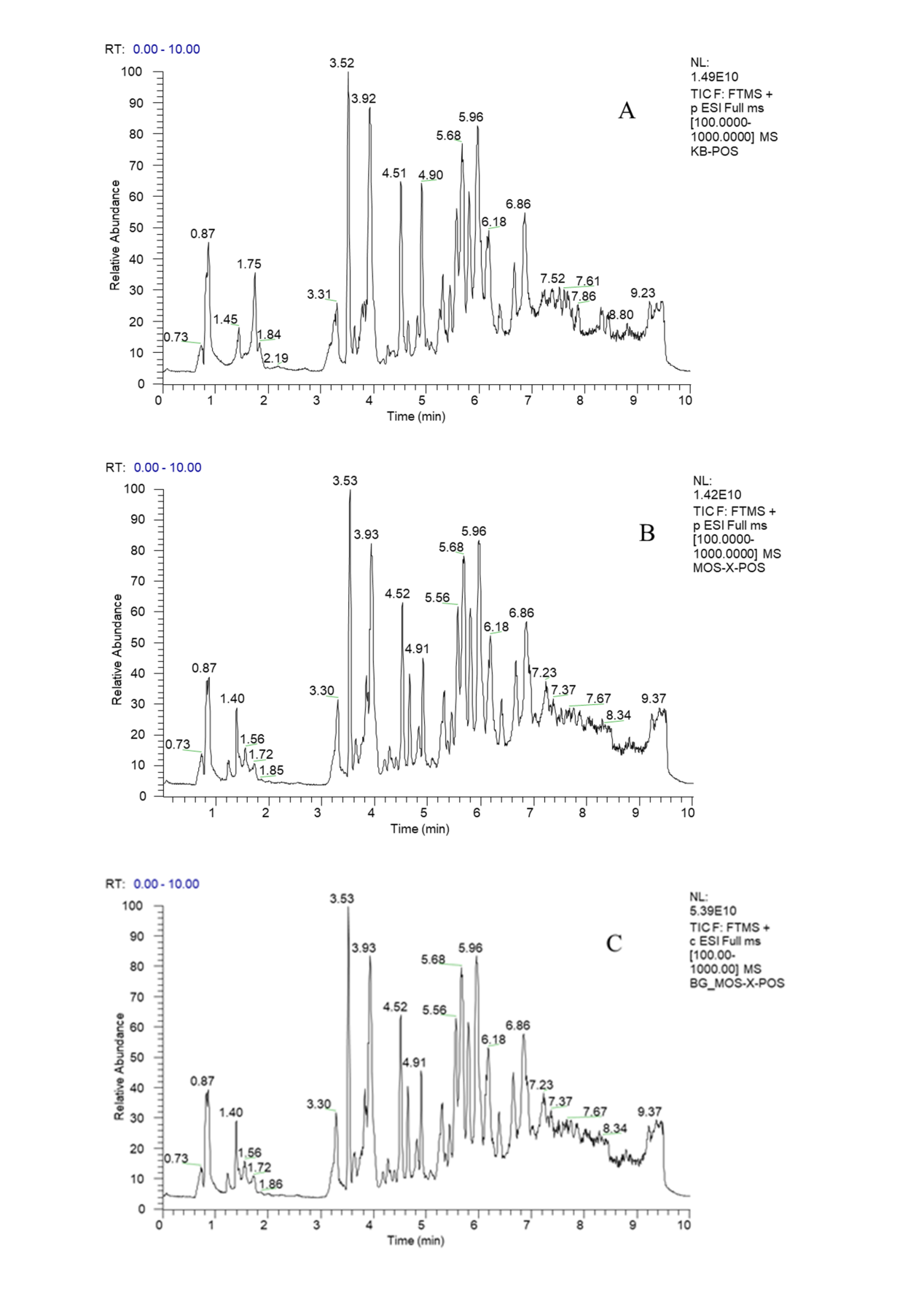
**

**Supplementary Figure 1. The total ions chromatographs of serum in positive ion mode.** (A) blank serum. (B) serum of MOS. (C) Difference in serum with MOS and blank.


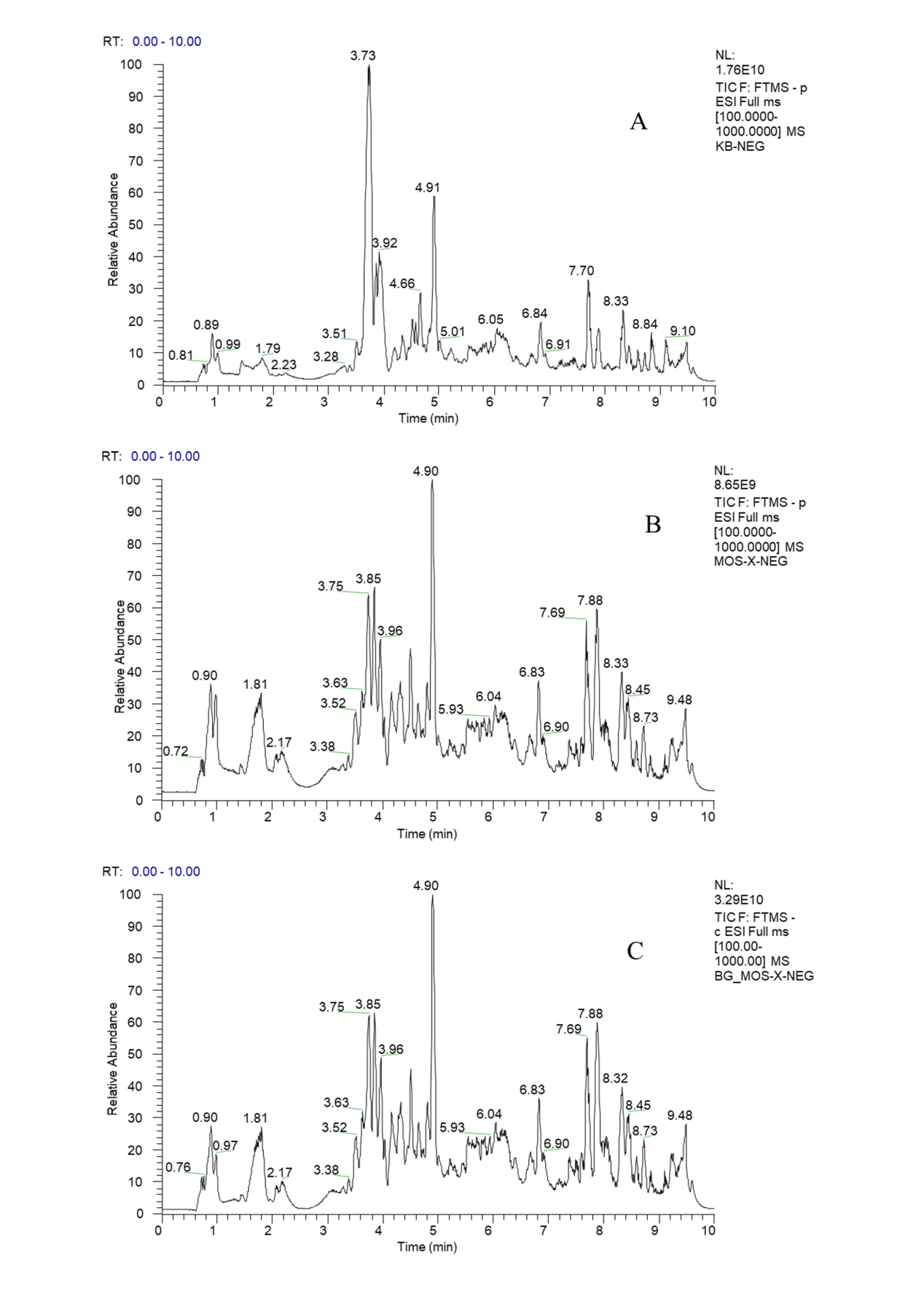


**Supplementary Figure 2. The total ions chromatographs of serum in negative ion mode.** (A) blank serum. (B) serum of MOS. (C) Difference in serum with MOS and blank.

**
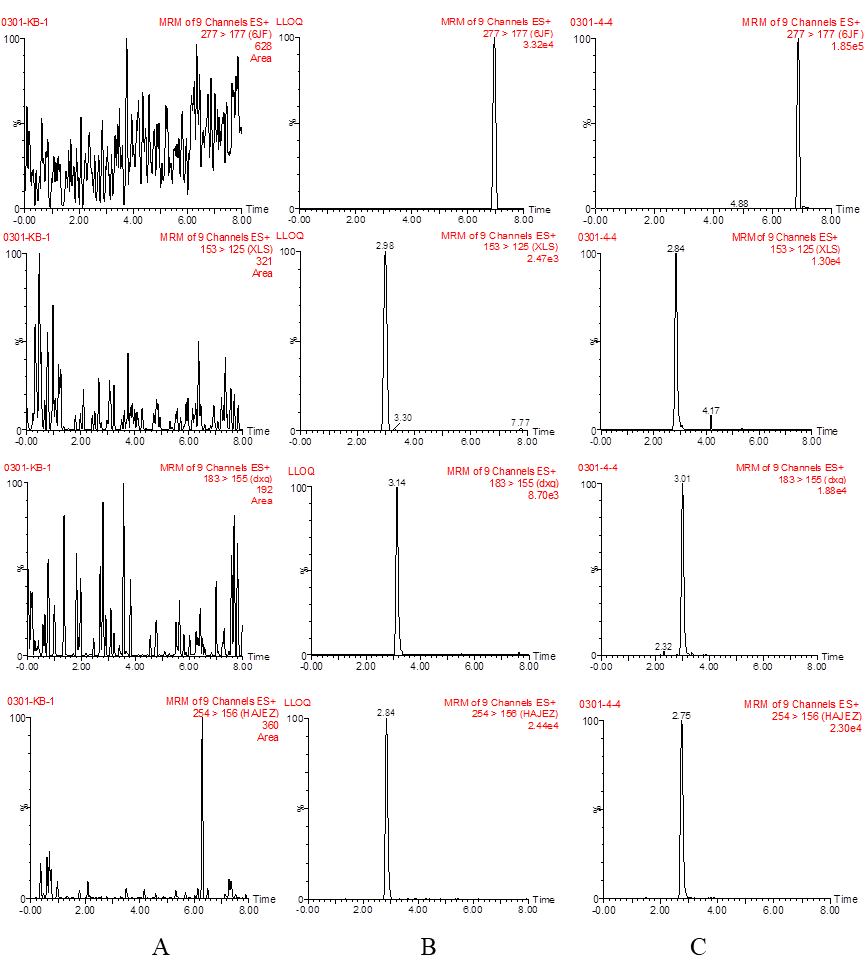
**

**Supplementary Figure 3.** Representative MRM chromatograms of (A) 6-Gingerol, Vanillin, Syringaldehyde and Sulfamethoxazole (IS) in rat plasma. blank plasma, (B) blank plasma spiked with 6-Gingerol, Vanillin and Syringaldehyde at LLOQ, (C) plasma sample obtained from a male rat 0.5 h after drug administration.

**Supplementary Table 1.** The regression equations, liner ranges and LLOQs for the determination of 6-Gingerol, Vanillin and Syringaldehyde.

| Compound | Regression Equation | *r* | Linear Range  (ng/mL) | LLOQ  (ng/mL) |
| --- | --- | --- | --- | --- |
| 6-Gingerol | y=0.016x+0.071 | 0.9956 | 3.57-1785 | 3.570 |
| Vanillin | y=0.006x+0.008 | 0.9914 | 1.772-886 | 1.772 |
| Syringaldehyde | y=0.022x+0.037 | 0.9945 | 4.056-2028 | 4.056 |

**Supplementary Table 2.** Precision and accuracy for the determination of 6-Gingerol, Vanillin and Syringaldehyde in rat plasma by LC-MS/MS（n=6）

| Compound | Norminal  Conc.(ng/mL) | Intra-Day | | | Inter-Day | | |
| --- | --- | --- | --- | --- | --- | --- | --- |
|  |  | Measured Conc.(ng/mL) | RE% | RSD% | Measured Conc.(ng/mL) | RE% | RSD% |
| 6-Gingerol | 8.93 | 8.92 | -0.1% | 7.5% | 8.99 | 0.7% | 8.6% |
|  | 133.9 | 122.5 | -8.5% | 5.3% | 125.6 | -6.2% | 6.9% |
|  | 1339 | 1327 | -0.9% | 5.0% | 1338 | 0.0% | 3.9% |
| Vanillin | 4.250 | 4.43 | 4.3% | 8.3% | 4.510 | 6.1% | 7.4% |
|  | 66.45 | 67.64 | 1.8% | 3.1% | 67.10 | 1.0% | 6.2% |
|  | 664.5 | 678.8 | 2.2% | 3.6% | 671.0 | 1.0% | 5.1% |
| Syringaldehyde | 10.14 | 10.4 | 2.6% | 9.7% | 10.14 | 1.6% | 7.9% |
|  | 152.1 | 154.5 | 1.6% | 4.7% | 148.5 | -2.4% | 5.6% |
|  | 1521 | 1529 | 0.6% | 3.8% | 1544 | 1.6% | 5.9% |

**Supplementary Table 3.** Matrix effect and extraction recovery for 6-Gingerol, vanillin and Syringaldehyde in rat plasma (n=6).

| Compound | Norminal  Conc.(ng/mL) | Matrix Effect | | Extraction Recovery | |
| --- | --- | --- | --- | --- | --- |
|  |  | Mean (%) | RSD% | Mean (%) | RSD% |
| 6-Gingerol | 8.93 | 88.8% | 8.7% | 80.6% | 5.5% |
|  | 133.9 | 97.7% | 5.9% | 86.9% | 5.1% |
|  | 1339 | 92.9% | 4.0% | 91.6% | 1.6% |
| Vanillin | 4.250 | 81.0% | 8.7% | 74.2% | 7.4% |
|  | 66.45 | 87.0% | 7.8% | 87.0% | 7.4% |
|  | 664.5 | 96.7% | 3.6% | 91.7% | 5.1% |
| Syringaldehyde | 10.14 | 84.6% | 5.4% | 88.6% | 10.5% |
|  | 152.1 | 97.9% | 9.5% | 79.4% | 4.6% |
|  | 1521 | 96.1% | 7.5% | 90.6% | 1.7% |

**Supplementary Table 4.** Stability of 6-Gingerol, Vanillin and Syringaldehyde under various conditions (n=3).

| Compound | Norminal Conc. (ng/mL) | Stability | | | | | | | |
| --- | --- | --- | --- | --- | --- | --- | --- | --- | --- |
|  |  | Room temperature for 24h | | Post-Preparation for 12h | | Three Freeze-Thaw cycles | | Storage at -80 ºC for 15 d | |
|  |  | RE% | RSD% | RE% | RSD% | RE% | RSD% | RE% | RSD% |
| 6-Gingerol | 8.93 | 5.9% | 8.3% | 6.8% | 9.2% | 1.2% | 4.3% | 3.4% | 2.9% |
|  | 1339 | 2.5% | 3.3% | 2.4% | 5.7% | 2.6% | 4.4% | 5.7% | 1.2% |
| Vanillin | 4.250 | 7.1% | 7.1% | 6.9% | 9.4% | 4.9% | 10.8% | 3.9% | 8.5% |
|  | 664.5 | -1.5% | 0.3% | -6.7% | 9.8% | -6.2% | 6.5% | 0.1% | 2.5% |
| Syringaldehyde | 10.14 | 6.8% | 8.8% | -2.0% | 2.1% | 1.3% | 3.1% | 4.5% | 8.5% |
|  | 1521 | -0.5% | 7.5% | -4.6% | 5.2% | 1.2% | 7.4% | 1.8% | 6.3% |

**Supplementary Table 5.** Pharmacokinetic parameters of the 6-Gingerol, Vanillin and Syringaldehyde in rats after oral administration of MOS extract (*mean±SD*, n=6)

| Parameters | 6-Gingerol | Vanillin | Syringaldehyde |
| --- | --- | --- | --- |
| C*_max_*（ng/L） | 512.3±108.1 | 101.2±26.9 | 306.4±57.7 |
| T*_1/2_*（h） | 2.75±0.67 | 2.54±0.82 | 3.39±2.30 |
| T*_max_*（h） | 0.50±0.03 | 0.33±0.13 | 0.28±0.11 |
| AUC*_(0-t)_*（ng/L*h） | 1490±417 | 200±38 | 416±100 |
| AUC*_(0-∞)_*（ng/L*h） | 1534±384 | 214±41 | 468±135 |
